# Anti-Angiogenic Efficacy of PSORI-CM02 and the Associated Mechanism in Psoriasis *In Vitro* and *In Vivo*

**DOI:** 10.3389/fimmu.2021.649591

**Published:** 2021-04-30

**Authors:** Yue Lu, Yuqi Yang, Junhong Zhang, Hongyu Zhang, Changju Ma, Xiaojuan Tang, Jingjing Wu, Li Li, Jianan Wei, Haiming Chen, Chuanjian Lu, Ling Han

**Affiliations:** ^1^ The Second Clinical Medical College, Guangzhou University of Chinese Medicine, Guangzhou, China; ^2^ State Key Laboratory of Dampness Syndrome of Chinese Medicine, The Second Affiliated Hospital of Guangzhou University of Chinese Medicine (Guangdong Provincial Hospital of Chinese Medicine), Guangzhou, China; ^3^ Guangdong Provincial Key Laboratory of Clinical Research on Traditional Chinese Medicine Syndrome, The Second Affiliated Hospital of Guangzhou University of Chinese Medicine, Guangzhou, China; ^4^ Guangdong-Hong Kong-Macau Joint Lab on Chinese Medicine and Immune Disease Research, Guangzhou University of Chinese Medicine, Guangzhou, China

**Keywords:** PSORI-CM02, psoriasis, angiogenesis, oxidative stress, inflammation, MAPK signalling pathway

## Abstract

Psoriasis is a chronic proliferative autoimmune dermatologic disease characterised by abnormal angiogenesis. Thus, regulating angiogenesis in the skin is an important treatment strategy for psoriasis. PSORI-CM02, an empirical Chinese medicine formula optimised from Yin Xie Ling, was created by the Chinese medicine specialist, Guo-Wei Xuan. Clinical studies have shown that PSORI-CM02 is safe and effective for the treatment of psoriasis. However, its anti-psoriatic mechanisms remain to be further explored. In this study, we investigated the effects of PSORI-CM02 on angiogenesis in the skin and the underlying mechanisms in IL-17A-stimulated human umbilical vein endothelial cells (HUVECs) and a murine model of imiquimod (IMQ)-induced psoriasis. *In vitro*, PSORI-CM02 significantly inhibited the proliferation and migration of IL-17A-stimulated HUVECs in a dose-dependent manner. Further, it markedly regulated the antioxidative/oxidative status and inflammation; suppressed the expression of VEGF, VEGFR1, VEGFR2, ANG1, and HIF-1α; and reduced the phosphorylation of MAPK signalling pathway components in IL-17A-stimulated HUVECs. *In vivo* studies showed that PSORI-CM02 markedly reduced angiogenesis in the skin of mice with IMQ-induced psoriasis, while significantly rebalancing antioxidant/oxidant levels; inhibiting the production of IL-6, TNF-α, IL-17A, and IL-17F; and repressing the synthesis of angiogenic mediators. In addition, PSORI-CM02 markedly reduced the activation of the MAPK signalling pathway in psoriatic skin tissue. Taken together, our results demonstrated that PSORI-CM02 inhibited psoriatic angiogenesis by reducing the oxidative status and inflammation, suppressing the expression of angiogenesis-related molecules, and inhibiting the activation of the MAPK signalling pathway *in vitro* and *in vivo*.

## Introduction

Psoriasis is a chronic autoimmune skin disorder that affects approximately 125 million people worldwide (1). Keratinocyte overproliferation, multiple immune cell infiltration, and hypervascular hyperplasia are the characteristic pathological manifestations of psoriasis (2). The development of psoriasis is related to various factors, including innate and adaptive immune responses, genetic factors, environmental factors, and metabolic disorders (3). Topical agents, including corticosteroids, vitamin D analogues, calcineurin inhibitors, and keratolytics, remain the cornerstone of treatment for patients with mild psoriasis (4). Biologics that inhibit tumour necrosis factor (TNF)-α, p40IL-12/23, interleukin (IL)-17, or p19IL-23 are an option for the first-line treatment of moderate to severe plaque psoriasis (5). However, new drugs and methods are needed to improve treatment efficacy (6). As angiogenesis is a key pathological feature in the development of psoriasis, antibodies and other types of molecules that exert anti-angiogenic effects are currently being evaluated and represent promising approaches to treatment (7).

An imbalance between oxidative and anti-oxidative molecules is an essential factor that leads to the development and aggravation of psoriasis. Elevated levels of oxidative stress result in the activation of Th1 cells, Th17 cells, and keratinocytes through the MAPK signalling pathway (8, 9). This results in the release of inflammatory cytokines and growth factors, including IL-1β, TNF-α, IL-17A, IL-17F, IL-6, vascular endothelial growth factor (VEGF), and angiopoietin-1 (ANG1) (10), which promote angiogenesis during the pathogenesis of psoriasis (11, 12). Components of the mitogen-activated protein kinase (MAPK) family include extracellular signal-regulated kinases (ERKs), c-Jun N-terminal kinases (JNKs), and p38 MAPK (13). Previous studies have shown that the activation of ERK1/2, p38 MAPK, and JNK is increased in psoriatic skin lesions (14, 15). The MAPK signalling pathway plays a crucial role in angiogenesis in psoriasis, and drugs designed to increase vascularity in psoriasis patients are closely related to this pathway (16).

PSORI-CM02 was optimised from an empirical prescription of Yin Xie Ling by Professor Guo-Wei Xuan, a national Chinese medicine expert specialising in dermatology. PSORI-CM02 consists of five herbal components: *Rhizoma Curcumae*, *Radix Paeoniae rubra*, *Sarcandra glabra*, *Rhizoma Smilacis glabrae*, and *Fructus mume* (17). The formula has been used to treat psoriasis at the Guangdong Provincial Hospital of Chinese Medicine for decades. Our previous study found that PSORI-CM02 inhibits the proliferation of HaCaT cells and reduces the psoriasis area and severity index scores in mice with imiquimod (IMQ)-induced psoriasis (17), regulates the T cell balance (17, 18), inhibits the phosphorylation of components of the PI3K/Akt/mTOR pathway to promote autophagy (19), inhibits the NF-κB signalling pathway and inflammatory factor secretion (17), and promotes M2-type macrophage polarisation (20).

In this study, we investigated the effects of PSORI-CM02 on angiogenesis in IL-17A-stimulated human umbilical vein endothelial cells (HUVECs) and a mouse model of IMQ-induced psoriasis. We further explored the anti-angiogenesis mechanism of PSORI-CM02 in the treatment of psoriasis, to provide a foundation for the clinical application of PSORI-CM02.

## Material and Methods

### Reagents

Minimal essential medium (MEM) and foetal bovine serum (FBS) were purchased from Gibco Laboratories (Grand Island, NY, USA; catalogue nos. A1049001 and 12483020, respectively). Axitinib was obtained from MedChemExpress (Monmouth Junction, NJ, USA; catalogue no. HY-10065). Recombinant human IL-17A was purchased from Peprotech (Rocky Hill, NJ, USA; catalogue no. 96-200-17-5). 3-(4, 5-Dimethylthiazol-2-yl)-2, 5-diphenyltetrazolium bromide (MTT) was obtained from Sigma-Aldrich (St. Louis, MO, USA; catalogue no. M2128). Assay kits for superoxide dismutase (SOD), reactive oxygen species (ROS), malonaldehyde (MDA), lactate dehydrogenase (LDH), glutathione (GSH), and catalase (CAT) and enzyme-linked immunosorbent assay (ELISA) kits for human VEGF and HIF-1α were purchased from Beyotime Biotechnology (Shanghai, China; catalogue nos. S0101, S0033M, S0131, C0016, S0053, S0051, PV963, and PH368, respectively). MTX was obtained from Shanghai Xinyi Pharmaceutical Factory (Shanghai, China; catalogue no. H31020644). IMQ cream was obtained from Sichuan Mingxin Pharmaceutical Co, Ltd (Sichuan, China; catalogue no. H20030128). TRIzol and cDNA synthesis kits were obtained from Invitrogen (Carlsbad, CA, USA; catalogue nos. 15596018 and 4374967, respectively). Reverse transcription-polymerase chain reaction (RT-PCR) primers were synthesised by Invitrogen (Shanghai, China). Antibodies specific for ERK1/2, p-ERK1/2 (phospho T202 + Y204), p38, p-p38 (phospho T180 + Y182), JNK1, p-JNK1 (phospho T183), VEGF receptor 1 (VEGFR1), VEGF receptor 2 (VEGFR2), HIF-1α, and ANG1 were acquired from Abcam (Cambridge, UK; catalogue nos. ab17942, ab278538, ab31828, ab4822, ab199380, ab47337, ab32152, ab2349, ab1, and ab8451, respectively). The anti-GAPDH antibody was obtained from Cell Signaling Technology (Danvers, MA, USA; catalogue no. 9145).

### The Preparation of PSORI-CM02

The plant species used in this study were *Rhizoma Curcumae*, *Radix Paeoniae Rubra*, *S. glabra*, *Rhizoma Smilacis glabrae*, and *Fructus mume*. All plant materials were pharmacopeia-grade and were obtained from Kangmei Pharmaceutical Company, Ltd. (Guangzhou, China). Extracts of the herbs were prepared in distilled water and were concentrated and stored at 4°C for the study. Ultra-performance liquid chromatography was used to monitor batches of the PSORI-CM02 formula for quality control purposes, as described previously (17, 19).

### IL-17A-Stimulated HUVECs

HUVECs were obtained from the Cell Culture Unit of the Shanghai Science Academy (Shanghai, China). They were cultured in MEM (Gibco, Waltham, MA, USA) with 10% FBS (Gibco) at 37°C and 5% CO_2_ according to the manufacturer’s instructions. The cells were randomly divided into the following groups: (1) control group; (2) IL-17A group; (3) IL-17A + low-dose/high-dose PSORI-CM02 group, in which the cells were treated with PSORI-CM02 (1.25 mg/mL or 2.5 mg/mL) for 24 h and then incubated with IL-17A (20 ng/mL) for another 6 h; and (4) IL-17A + axitinib group, in which the cells were treated with axitinib (0.1 nM) for 24 h and then incubated with IL-17A (20 ng/mL) for another 6 h.

### MTT Assay

Cell viability was measured using an MTT reduction assay. The cells were seeded into a 96-well plate in MEM + 10% FBS at a density of 5,000 cells per well. PSORI-CM02 or MEM (control) was added to the wells, and the cells were incubated for 24 or 48 h. IL-17A was then added, and the cells were incubated for a further 6 h. Subsequently, 10 μL of MTT solution was added to each well, and the cells were incubated for 4 h in a 37°C incubator. Finally, the purple crystals were lysed with 100 μL of 0.04 N HCl in isopropyl alcohol, and the absorbance of each well at 570 nm was measured.

### Transwell Cell Migration Assay

Serum-starved HUVECs were treated with or without PSORI-CM02 (1.25 mg/mL or 2.5 mg/mL) or axitinib for 24 h at 37°C, and were then added to the upper chamber of a 24-well plate containing transwell inserts with a pore size of 8.0 μm (Corning Inc., Corning, NY, USA). IL-17A (20 ng/mL) in MEM containing 20% FBS was added to the lower chamber. After 6 h of incubation at 37°C in a CO_2_ incubator, wet cotton swabs were used to remove the non-migrating cells from the membrane. The membrane was then fixed with 4% paraformaldehyde for 10 min, stained with 0.1% crystal violet for 10 min, and washed with distilled water. The cells were observed using an IX70 fluorescence microscope (Olympus, Tokyo, Japan).

### Animals

Male BALB/c mice (weighing 18-22 g) at 6-8 weeks of age were purchased from the Experimental Animal Center of Guangdong Province (Guangzhou, China). Mice were given free access to water and fed a standard diet under standard laboratory conditions. All animal protocols were approved by the Animal Experimental Ethics Committee of Guangdong Provincial Hospital of Chinese Medicine.

### Imiquimod-Induced Psoriasis-Like Mouse Model

A daily topical dose of 50 mg of IMQ cream was applied to a shaved area (3 × 2.5 cm) on the back of each mouse for 7 consecutive days to create a psoriasis-like mouse model, as previously described (19).

### PSORI-CM02 Administration

Thirty BALB/c mice were randomly divided into five groups: (1) the control and IMQ groups received distilled water orally; (2) the IMQ + MTX group received 1 mg/kg of MTX orally; (3) the IMQ + medium-dose PSORI-CM02 group received 1.42 g/kg of PSORI-CM02 orally; and (4) the IMQ + high-dose PSORI-CM02 group received 2.84 g/kg of PSORI-CM02 orally. Drug administration began 4 h before the daily application of IMQ cream and continued for 7 days.

### Measurements of SOD, ROS, MDA, LDH, GSH and CAT

HUVECs or skin tissues were homogenised in Tris buffer (20 mM, pH 7.5) on ice using an Ultra-Turrax homogeniser (IKA, Staufen, Germany) and centrifuged at 12,000 rpm at 4°C for 10 min. The resulting supernatant was used to measure the activities or levels of SOD, ROS, MDA, LDH, GSH, and CAT using commercial assay kits, following the manufacturer’s instructions.

### ELISAs

HUVECs were collected, and the expression levels of VEGF and HIF-1α were assessed using ELISA kits, according to the manufacturer’s instructions. The absorbance of each well was measured at 450 nm.

### Real-Time PCR Analysis

PSORI-CM02-treated HUVECs were harvested 6 h after the addition of IL-17A to analyse the expression of *IL-1β*, *TNF-α*, *IL-6*,and *GAPDH* mRNAs. Skin tissues were also collected from mice to analyse the mRNA expression levels of *IL-6*, *TNF-α*, *IL-17A*, *IL-17F*, *VEGF*, *HIF-1α*, and *GAPDH*. Total RNA was isolated using TRIzol reagent. RNA purity and concentrations were assessed using a NanoDrop 2000 instrument (Thermo Fisher Scientific, Waltham, MA, USA). RNA samples were then reverse-transcribed into cDNA using an RT-PCR kit, according to the manufacturer’s instructions. The PCR amplification conditions involved an initial denaturation step at 95°C for 15 s, followed by 35 cycles of denaturation at 95°C for 5 s and annealing at 61°C for 15 s. Real-time PCR was performed using SYBR Premix Ex TaqTM II (Takara, Kusatsu, Japan) and a ViiA7 real-time PCR instrument (Thermo Fisher Scientific). The sequences of the respective human sense and antisense primers were as follows (from 5′ to 3′): *IL-6*, CCACCGGGAACGAAAGAGAA and TCTTCTCCTGGGGGTACTGG; *IL-1β*, GTTCCCTGCCCACAGACCT and TGGACCAGACATCACCAAGC; *TNF-α*, CACGCTCTTCTGCCTGCT and GCTTGTCACTCGGGGTTC; and *GAPDH*, TGTGGGCATCAATGGATTTGG and ACACCATGTATTCCGGGTCAAT. The sequences of the respective mouse sense and antisense primers were as follows (from 5′ to 3′): *IL-6*, GAGGATACCACTCCCAACAGACC and AAGTGCATCATCGTTGTTCATACA; *TNF-α*, GGGTGTTCATCCATTCTCTACC and GTCCCAG-CATCTTGTGTTTC; *IL-17A*, CTGACCCCTAAGAAACCCC and GAAGCAGTTTGGGACCCCTT; *IL-17F*, ACGTGAATTCCAGAACCGCTA and TGATGCAGCCTGAGTGTCTG; *VEGF*, TATTCAGCGGACTCACCAGC and AACCAACCTCCTCAAACCGT; *HIF-1α*, CTTGACAAGCTAGCCGGAGG and TCGACGTTCAGAACTCATCCT; and *GAPDH*, AAGAGGGATGCTGCCCTTAC and TACGGCCAAATCCGTTCACA. Relative mRNA quantities were determined using the 2^-ΔΔCt^ method, with data normalised to the *GAPDH* housekeeping gene.

### Western Blotting

Homogenised cells or tissues were lysed in lysis buffer (50 mM Tris [pH 7.4], 150 mM NaCl, 0.1% sodium dodecyl sulphate, 1% sodium deoxycholate, 1 mmol/L phenylmethylsulphonyl fluoride, 1% Triton X-100, and protease inhibitors), and then centrifuged at 15,000 rpm at 4°C for 15 min to obtain total protein samples. Total cellular protein concentrations were quantified using a BCA Protein Assay Kit (Thermo Fisher Scientific), according to the manufacturer’s instructions. The proteins in each sample were resolved by 12.5% sodium dodecyl sulphate-polyacrylamide gel electrophoresis and transferred onto polyvinylidene difluoride membranes. The membranes were blocked with 3% bovine serum albumin at room temperature for 30 min. The blocked membranes were then incubated with various primary antibodies at 4°C overnight, followed by 1 h of incubation with various secondary antibodies. Finally, the proteins were detected using a Bio-Rad Imaging System (Bio-Rad Biosciences, Hercules, CA, USA).

### Immunochemical Analysis

Formalin-fixed skin specimens were embedded in paraffin and sectioned at a thickness of 7 μm. The sections were dewaxed by heating at 55°C for 30 min, washing twice for 15 min each, rehydrating in ethanol for 15 min, and incubating in water at 95°C for 5 min. Antigens were retrieved by treating the tissue sections with 3% hydrogen peroxide for 30 min. The sections were then incubated at 4°C overnight with an anti-VEGF antibody diluted to 1:100, an anti-aANG1 antibody diluted to 1:100, or an anti-HIF-1α antibody diluted to 1:50. The sections were then washed with phosphate-buffered saline (PBS) and incubated with the secondary antibody at 37°C for 30 min. 3′3-Diaminobenzidine tetrahydrochloride was added to detect the protein-antibody complexes. Negative control sections were incubated with PBS instead of the primary antibody. The expression levels of VEGF, angiopoietin 1, and HIF-1α were quantified using Image-Pro Plus software (Media Cybernetics, Rockville, MD, USA).

### Statistical Analysis

Data are presented as the means ± SEM. Student’s t-test was used to assess differences between two independent groups. A one-way analysis of variance was used for comparisons between three or more groups, with the Bonferroni correction for multiple comparisons. Statistical analyses were performed using SPSS 19.0 software (IBM, Armonk, NY, USA), and data were visualised using Prism 5.0 (GraphPad, San Diego, CA, USA). A *p* value less than 0.05 was considered to indicate statistical significance. At least three independent experiments were performed.

## Results

### PSORI-CM02 Inhibits the Proliferation and Migration of IL-17A-Stimulated HUVECs

**Figure 1:**
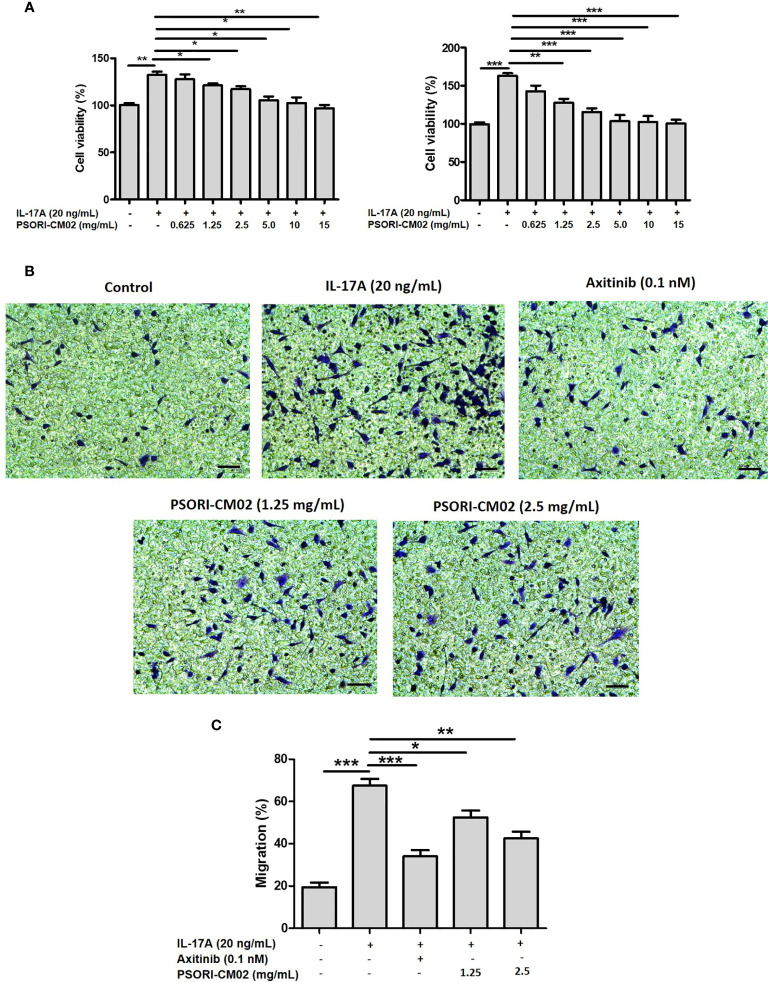
PSORI-CM02 inhibited the proliferation and migration of interleukin (IL)-17A-stimulated human umbilical vein endothelial cells (HUVECs). **(A)** HUVECs were treated with various concentrations of PSORI-CM02 from 0.625 to 15 mg/mL for 24 h and 48 h, and then stimulated with 20 ng/mL IL-17A for 6 h. The effects of PSORI-CM02 on IL-17A-stimulated cell viability were assessed *via* MTT assay. **(B)** Light microscope photographs indicate cell mobility in the transwell migration experiment (×100 magnification, scale bar = 50 μm). **(C)** Quantification of the HUVEC migration. Data represent the mean ± SEM of at least three independent experiments (*p < 0.05, **p < 0.01, ***p < 0.001 *vs*. the IL-17A-stimulated HUVEC group).

IL-17 induces angiogenesis by promoting the secretion of HIF-1α and VEGF and the migration of vascular endothelial cells (21). Therefore, we used IL-17A-stimulated HUVECs as an *in vitro* angiogenesis model (22) to explore the anti-angiogenic effects of PSORI-CM02 and the associated mechanisms. We first evaluated the effects of PSORI-CM02 on the proliferation of IL-17A-stimulated HUVECs. The viability of IL-17A-stimulated HUVECs was reduced in a dose-dependent manner after 24 and 48 h of exposure to various concentrations of PSORI-CM02 (0.625, 1.25, 2.5, 5, 10, and 15 mg/mL; **Figure 1A**). Axitinib can effectively inhibit angiogenesis and was thus chosen as the positive control drug for the *in vitro* experiments (23). The migration of IL-17A-stimulated HUVECs was significantly inhibited after PSORI-CM02 treatment (**Figures 1B, C**), indicating that HUVEC migration was promoted by IL-17A treatment, but markedly inhibited by PSORI-CM02 treatment.

### The Effects of PSORI-CM02 on SOD, GSH, CAT, ROS, MDA, LDH, IL-1β, TNF-α, and IL-6 Levels in IL-17A-Stimulated HUVECs

**Figure 2:**
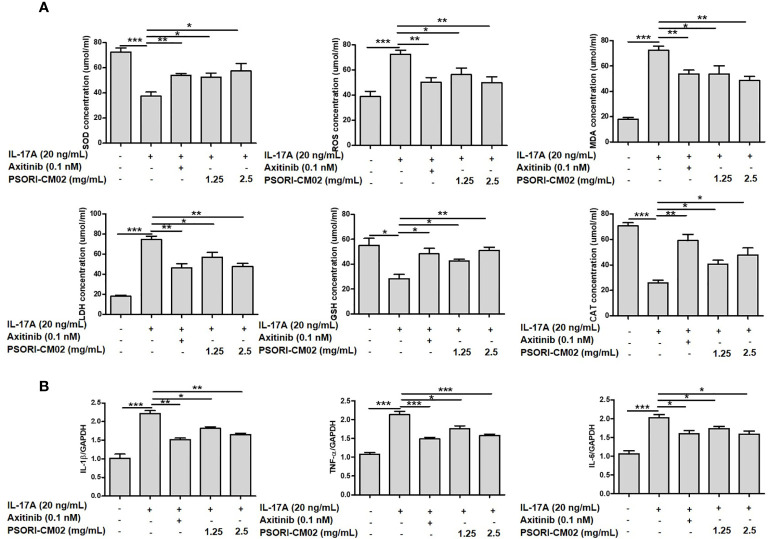
Effects of PSORI-CM02 on oxidative stress and inflammation in interleukin (IL)-17A-stimulated human umbilical vein endothelial cells (HUVECs). **(A)** HUVECs were treated with 1.25 or 2.5 mg/mL PSORI-CM02 for 24 h and then stimulated with 20 ng/mL IL-17A for 6 h. Subsequently, the cells were collected and tested using superoxide dismutase (SOD), reactive oxygen species (ROS), malondialdehyde (MDA), lactate dehydrogenase (LDH), glutathione (GSH), and catalase (CAT) assay kits, according to the manufacturers’ instructions, to detect the effects of PSORI-CM02 on antioxidative/oxidative factors. **(B)** Total RNA was isolated from HUVECs, and RT-PCR was used to investigate the levels of various pro-inflammatory cytokines. Data represent the mean ± SEM of at least three independent experiments (*p < 0.05, **p < 0.01, ***p < 0.001 *vs.* the IL-17A-stimulated HUVEC group).

Upregulated levels of oxidative factors accelerate the activation of T cells and keratinocytes, which promotes the synthesis and release of inflammatory cytokines and growth factors, consequently contributing to hypervascular hyperplasia in psoriasis (24). As oxidative stress and inflammation involves angiogenesis, we investigated changes in antioxidative/oxidative factors and inflammatory cytokines in IL-17A-stimulated HUVECs. As shown in **Figure 2A**, treatment with PSORI-CM02 significantly up-regulated the activities of SOD, GSH, and CAT and downregulated the levels of ROS, MDA, and LDH in IL-17A-stimulated HUVECs. The IL-1β, TNF-α, and IL-6 secretion levels were upregulated in IL-17A-stimulated HUVECs compared with control cells, whereas PSORI-CM02 markedly downregulated the mRNA levels of *TNF-α* and *IL-6*, as determined by RT-PCR (**Figure 2B**).

### PSORI-CM02 Suppresses the Expression of Angiogenic Mediators and the Phosphorylation of MAPK Signalling Pathway Components in IL-17A-Stimulated HUVECs

**Figure 3:**
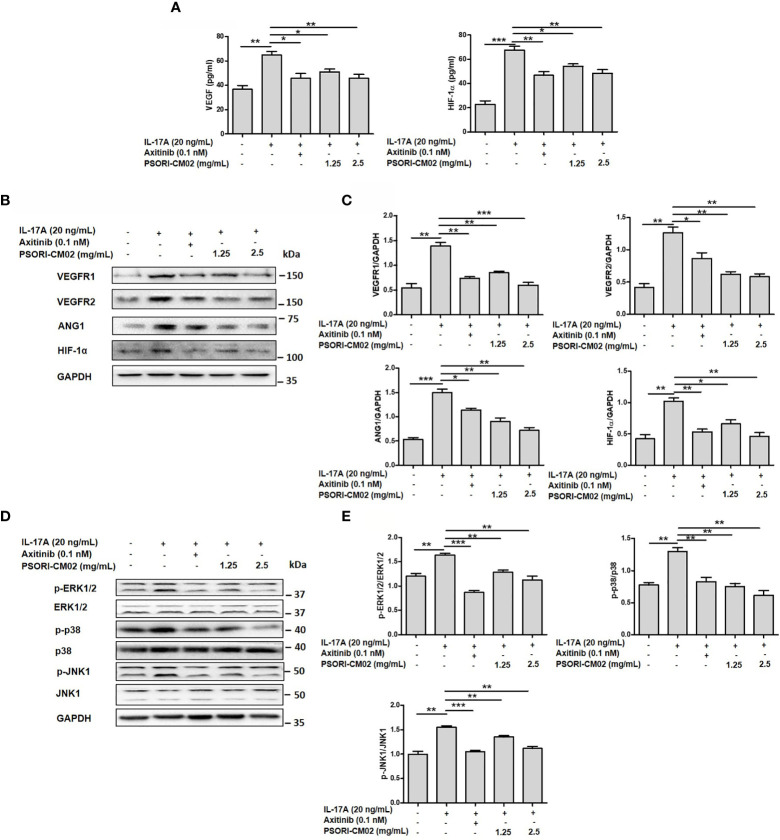
PSORI-CM02 suppressed the expression of pro-angiogenic factors and the phosphorylation of MAPK signalling pathway components in interleukin (IL)-17A-stimulated human umbilical vein endothelial cells (HUVECs). **(A)** HUVECs were treated with 1.25 or 2.5 mg/mL PSORI-CM02 for 24 h and then stimulated with 20 ng/mL IL-17A for 6 h. The levels of hypoxia inhibitory factor (HIF)-1α and vascular endothelial growth factor (VEGF) were determined by ELISA. **(B)** Representative western blots of VEGF receptor 1 (VEGFR1), VEGFR2, angiopoietin 1 (ANG1), and HIF-1α expression in HUVECs treated with 1.25 and 2.5 mg/mL PSORI-CM02 or 0.1 nM axitinib for 24 h and then stimulated with 20 ng/mL IL-17A for 6 h. **(C)** Quantification of *VEGFR1*, *VEGFR2*, *ANG1*, and *HIF-1α* levels relative to *GAPDH* levels at 24 h. **(D)** Representative western blot of MAPK pathway protein expression in HUVECs treated with 1.25 and 2.5 mg/mL PSORI-CM02 or 0.1 nM axitinib for 24 h and then stimulated with 20 ng/mL IL-17A for 6 h. **(E)** Quantification of phospho-ERK1/2, phospho-p38, and phospho-JNK1 relative to ERK1/2, p38, and JNK1 at 24 h. Data represent the mean ± SEM of at least three independent experiments (*p < 0.05, **p < 0.01, ***p < 0.001 *vs.* the IL-17A- stimulated HUVEC group).

Several angiogenic mediators, such as VEGF, VEGFR, HIF-1α, and ANG1, are upregulated in psoriatic lesions to promote the formation of new blood vessels (25). To evaluate the effects of PSORI-CM02 on pro-angiogenic factors in IL-17A-stimulated HUVECs, we measured the expression levels of VEGF, VEGFR1, VEGFR2, ANG1, and HIF-1α. As shown in **Figures 3A–C**, the levels of these molecules were upregulated significantly in IL-17A-stimulated HUVECs compared with the control cells, whereas PSORI-CM02 markedly downregulated the levels of angiogenesis-related markers, as indicated by ELISA and western blotting results. As the classical MAPK pathway is closely involved in the regulation of angiogenesis (14), we investigated the MAPK signalling pathway in IL-17A-stimulated HUVECs. Treatment with PSORI-CM02 significantly inhibited the phosphorylation of ERK1/2, p38, and JNK1 (**Figures 3D, E**) in IL-17A-stimulated HUVECs. Therefore, PSORI-CM02 may inhibit the MAPK signalling pathway to inhibit angiogenesis.

### PSORI-CM02 Alleviates Angiogenic Manifestations and Reduces Oxidative Stress and Inflammation in the Skin of Mice With IMQ-Induced Psoriasis

**Figure 4:**
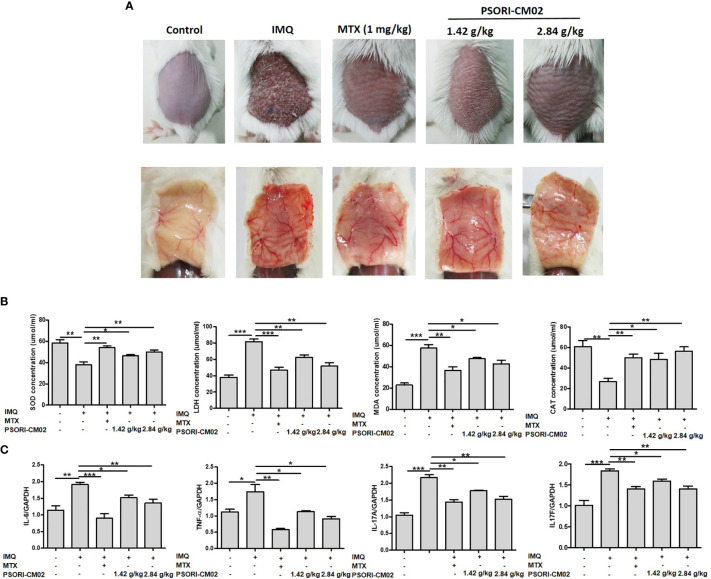
PSORI-CM02 inhibited angiogenesis in mice with imiquimod (IMQ)-induced psoriasis. **(A)** Photographs of the backs of mice 7 days after the first IMQ application. Mice were randomly divided into five groups, and methotrexate (MTX) or PSORI-CM02 administration began 4 h before daily IMQ cream application for 7 days. **(B)** Superoxide dismutase (SOD), lactate dehydrogenase (LDH), malondialdehyde (MDA), and catalase (CAT) levels were measured in back skin tissues of mice from each group using commercial assay kits. **(C)** The mRNA levels of interleukin (IL)-6 (*IL-6*), tissue necrosis factor-α (*TNF-α*), *IL-17A*, and *IL-17F* in mouse skin tissues were determined by RT-PCR. Data represent the mean ± SEM of at least three independent experiments (*p < 0.05, **p < 0.01, ***p < 0.001 *vs.* the IMQ-induced psoriasis group; n = 6).

The anti-angiogenic efficacy of orally administered PSORI-CM02 for the treatment of IMQ-induced psoriasis was evaluated in a mouse model. Methotrexate (MTX) is the main orally administered treatment for psoriasis (26) and was thus chosen as the positive control drug in this study. To visually represent the effect of PSORI-CM02 on angiogenesis at the lesion site, we photographed the back skin of mice on the 7th day of IMQ treatment. As shown in **Figure 4A**, compared with the control group, the extent of neovascularisation and vascular tortuousness were significantly increased in the IMQ-only group. However, the PSORI-CM02-treated groups showed a significant reduction in neovascularisation of the skin in mice with IMQ-induced psoriasis.

To determine the mechanism responsible for the effect of PSORI-CM02 on skin angiogenesis, we analysed antioxidative and oxidative molecules in the skin of mice with IMQ-induced psoriasis. As shown in **Figure 4B**, compared with the control group, SOD and CAT activities were significantly reduced in the skin of mice in the IMQ group, while the LDH and MDA levels were increased. SOD and CAT activities were higher in the IMQ + high-dose PSORI-CM02 group than the IMQ-only group, whereas the LDH and MDA levels were markedly lower in the IMQ + high-dose PSORI-CM02 group. These results showed that PSORI-CM02 balanced the levels of antioxidative and oxidative factors in the skin of mice with IMQ-induced psoriasis.

Several pro-angiogenic cytokines, such as IL-6, TNF-α, and IL-17, are up-regulated in psoriatic lesions, which may contribute to the increase in blood vessel formation in psoriasis patients (27). Therefore, we used RT-PCR to assess the mRNA levels of inflammatory cytokines, such as *IL-6*, *TNF-α*, *IL-17A*, and *IL-17F*, in skin tissues of mice with IMQ-induced psoriasis. As shown in **Figure 4C**, the levels of all four cytokines were upregulated in the skin of mice with psoriasis compared with the levels in the skin of control mice. However, PSORI-CM02 markedly downregulated the levels of these pro-inflammatory cytokines.

### PSORI-CM02 Inhibits the Synthesis of Angiogenic Factors in the Skin of Mice With IMQ-Induced Psoriasis

**Figure 5:**
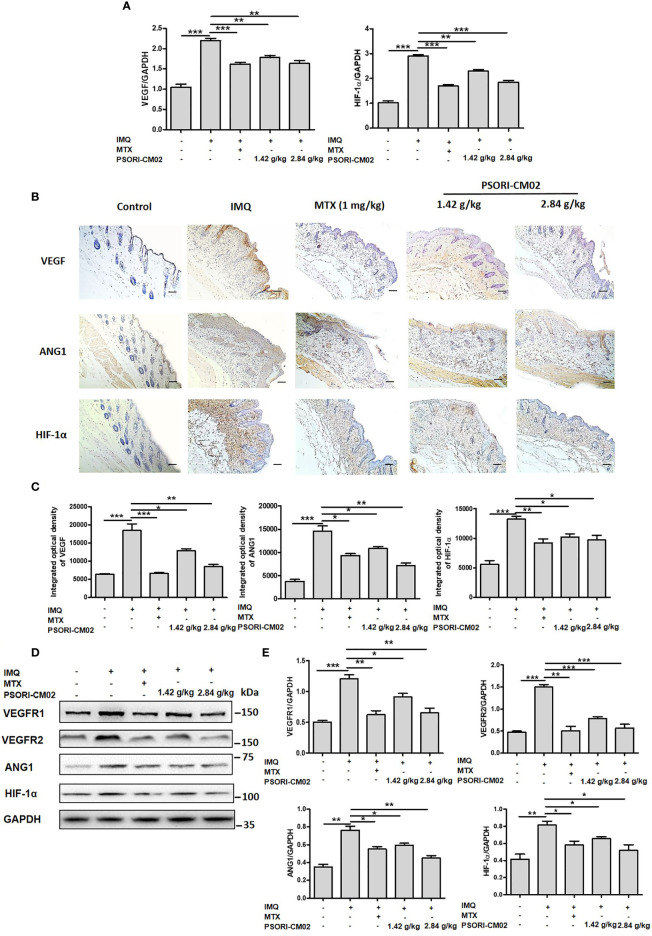
Effects of PSORI-CM02 on pro-angiogenic factors in mice with imiquimod (IMQ)-induced psoriasis. **(A)** Total RNA was isolated from skin tissues of mice, and RT-PCR was used to determine the levels of vascular endothelial growth factor (*VEGF*) and hypoxia inhibitory factor 1α (*HIF-1α*) mRNA. **(B)** Representative immunochemical images of VEGF, angiopoietin 1 (ANG1), and HIF-1α in skin tissue sections (×200 magnification, scale bar = 50 μm). **(C)** Quantification of integrated optical density of VEGF, ANG1, and HIF-1α in skin tissue sections. **(D)** Representative western blots of VEGF receptor 1 (VEGFR1), VEGFR2, ANG1, and HIF-1α protein expression in the skin of mice with IMQ-induced psoriasis. **(E)** Quantification of VEGFR1, VEGFR2, ANG1, and HIF-1α relative to GAPDH in the skin of mice (*p < 0.05, **p < 0.01, ***p < 0.001 *vs*. the IMQ-induced psoriasis group; n = 6).

VEGF is a significant mediator of angiogenesis and plays a key role in the formation of vascular abnormalities in psoriatic skin lesions (28). HIF-1α promotes the expression of VEGF (29), while ANG1 and VEGFR play essential roles in angiogenesis in psoriasis (30). To explore the anti-angiogenic mechanisms of PSORI-CM02, we further analysed the expression of *VEGF*, *VEGFR1*, *VEGFR2*, *ANG1*, and *HIF-1α*. As shown in **Figure 5A**, PSORI-CM02 treatment reduced the mRNA expression levels of *VEGF* and *HIF-1α* in the skin of mice with IMQ-induced psoriasis.

Next, we analysed VEGF and ANG1 expression by immunohistochemistry. Compared with control mice, IMQ-treated showed a greater area of brown precipitate in the epidermis. However, after the administration of PSORI-CM02, the area of brown precipitate in the epidermis significantly decreased. Immunohistochemical analysis of HIF-1α showed that PSORI-CM02 treatment reduced the area of brown precipitate in both the epidermis and the dermis, compared with the area of brown precipitate in IMQ-treated mice. The above results show that the protein expression levels of VEGF, ANG1, and HIF-1α decreased after PSORI-CM02 treatment (**Figures 5B, C**). Western blotting analysis also showed that PSORI-CM02 treatment significantly downregulated the expression levels of VEGFR1, VEGFR2, ANG1, and HIF-1α in mice with IMQ-induced psoriasis (**Figures 5D, E**). These results indicate that PSORI-CM02 treatment inhibits angiogenesis in psoriatic skin lesions induced by IMQ.

### PSORI-CM02 Suppresses the Activation of the MAPK Signalling Pathway in the Skin of Mice With IMQ-Induced Psoriasis

**Figure 6:**
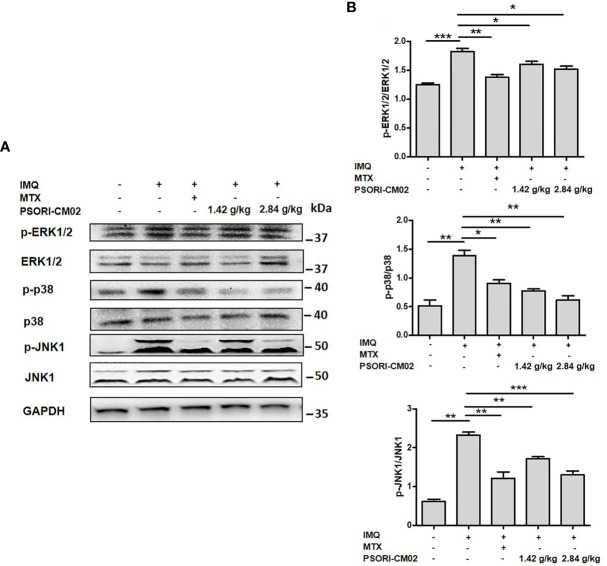
Effects of PSORI-CM02 on the MAPK signalling pathway in mice with (IMQ)-induced psoriasis. **(A)** Representative western blot of MAPK pathway protein expression in the skin of mice with IMQ-induced psoriasis. **(B)** Quantification of phospho-ERK1/2, phospho-p38, and phospho-JNK1 relative to ERK1/2, p38, and JNK1 in the skin of mice (*p < 0.05, **p < 0.01, ***p < 0.001 *vs.* the IMQ-induced psoriasis group; n = 6).

The MAPKs are a family of serine-threonine protein kinases that are involved in cell proliferation, apoptosis, differentiation, oxidative stress, signal transduction, and immune responses (31). The inhibition of MAPK signalling pathways down-regulates oxidative stress and inflammatory cytokine levels, thereby inhibiting angiogenesis in psoriasis (15). To investigate the anti-angiogenic mechanisms of PSORI-CM02 in IMQ-induced psoriasis, the phosphorylation levels of ERK1/2, p38, and JNK1 were examined using western blotting. As shown in **Figures 6A, B**, the phospho-ERK1/2:ERK1/2 ratio in skin tissues markedly decreased after PSORI-CM02 treatment. In addition, PSORI-CM02 significantly inhibited the phosphorylation of p38 and JNK1. Therefore, PSORI-CM02 inhibits the MAPK signalling pathway to play an anti-angiogenic role in IMQ-induced psoriasis.

## Discussion

Abnormal angiogenesis of skin lesions is a crucial pathological feature of psoriasis. The up-regulation of angiogenic mediators, including VEGF, HIF-1α, and ANG1, and pro-angiogenic cytokines, such as IL-17, TNF-α, IL-6, and IL-1β, contribute to this abnormality. These factors exert a synergistic effect on the development of psoriasis (8). Disruption of the oxidative stress-inflammation-angiogenesis axis leads to hypervascular hyperplasia in psoriasis (9, 10). The target of anti-angiogenesis therapy in psoriasis is closely related to this axis. Thus, we used IL-17A to stimulate HUVECs for *in vitro* experiments. Mice with IMQ-induced psoriasis have clinical manifestations and histopathological and immunological changes similar to human psoriasis vulgaris (32). Therefore, in this study, an IMQ-induced mouse model of psoriasis was used to assess the anti-angiogenic effects of PSORI-CM02 *in vivo*.

Previous studies by our group have shown that PSORI-CM02 ameliorates the skin symptoms of IMQ-induced psoriasis by regulating T cell balance and reducing inflammatory cytokine secretion (17, 18), promoting M2-type macrophage polarisation (20), and inhibiting the phosphorylation of components of the PI3K/Akt/mTOR pathway to promote epidermal autophagy (19). In this study, we investigated whether the anti-psoriatic effects of PSORI-CM02 depend on its anti-angiogenic effects *in vivo* and *in vitro* and explored the underlying mechanisms.

Abnormal microangiogenesis is closely related to the development of psoriasis (33). Microcirculation disturbances and hemorheological changes are observed in the skin lesions of psoriatic patients (34). In this study, we found that PSORI-CM02 inhibited the proliferation and migration of IL-17A-stimulated HUVECs and reduced new blood vessel growth in the skin of mice with IMQ-induced psoriasis.

Pro-angiogenic factors derived from keratinocytes and immune cells, such as oxidative molecules, antioxidative molecules, and inflammatory cytokines, contribute to blood vessel formation in psoriasis (35). Excessive levels of oxidative stress cause the secretion of inflammatory cytokines and growth factors, which induce vascular endothelial cell dysfunction (24) and can, consequently, contribute to angiogenesis. We detected a variety of antioxidative/oxidative molecules in IL-17A-stimulated HUVECs and the skin lesions of mice with IMQ-induced psoriasis. We found that PSORI-CM02 regulated the synthesis of antioxidative/oxidative molecules and inflammatory cytokines. These results suggested that PSORI-CM02 exerts anti-angiogenic effects by reducing the levels of oxidative stress and inflammation.

VEGF and its high-affinity tyrosine kinase receptors, VEGFR1 and VEGFR-2, are essential for vascular embryogenesis and neovascularisation (36, 37). *In situ* hybridisation and immunohistological analyses have shown that VEGF is upregulated in epidermal keratinocytes and VEGFR1 and VEGFR2 are overexpressed in psoriatic skin lesions (38, 39). HIF-1α, VEGF, and VEGFR play crucial roles in the onset and progression of psoriasis and are, therefore, promising targets for the treatment of psoriasis (40). In our study, PSORI-CM02 suppressed the expression of angiogenic mediators, including VEGF, HIF-1α, VEGFR1, VEGFR2, and ANG1, in IL-17A-stimulated HUVECs and in mice with IMQ-induced psoriasis.

The MAPK signalling cascade is a key pathway that regulates a wide variety of cellular processes, including proliferation, differentiation, apoptosis, and angiogenesis (41). The phosphorylation levels of ERK1/2, p38, and JNK1 are increased in psoriatic lesions, and this mediates the increase in oxidative stress and inflammation (42). Our results showed that PSORI-CM02 inhibited the phosphorylation of ERK1/2, p38, and JNK1 in IL-17A-stimulated HUVECs and in mice with IMQ-induced psoriasis, which suggests that the anti-angiogenic effects of PSORI-CM02 may be related to inhibition of the MAPK pathway. Our next study will focus on whether the PSORI-CM02-mediated inhibition of the phosphorylation of MAPK pathway components suppresses angiogenesis *in vivo* and *in vitro*.

In conclusion, the results of our study demonstrate that PSORI-CM02 inhibits the proliferation and migration of IL-17A-stimulated HUVECs. In mice with IMQ-induced psoriasis, PSORI-CM02 reduces neovascularisation and vascular tortuousness in skin lesions. *In vitro* and *in vivo*, PSORI-CM02 supresses the phosphorylation of the MAPK pathway, regulates the balance between oxidants and antioxidants, and decreases the release of inflammatory cytokines and the synthesis of angiogenic mediators. These anti-angiogenic effects may be the mechanism by which PSORI-CM02 effectively treats psoriasis. Further investigations are required to assess the relationship between PSORI-CM02 and the MAPK pathway, oxidative stress, inflammation, and angiogenesis *in vivo* and *in vitro*.

## Data Availability Statement

The original contributions presented in the study are included in the article/supplementary material, further inquiries can be directed to the corresponding authors.

## Ethics Statement

The animal study was reviewed and approved by Animal Welfare and Ethics Branch of the Biomedical Ethics Committee of Guangzhou University of Chinese Medicine.

## Author Contributions

LH and CL conceived and designed the experiments. YL, YY, JZ, HZ, CM, and XT performed the experiments. YL, YY, JWu, LL, JWei, and HC analysed and interpreted the data. LH and CL performed data analysis and interpretation. YL, YY, and JZ wrote the article. All authors contributed to the article and approved the submitted version.

## Funding

This research was financially supported by the National Natural Science Foundation of China (82004363 and 81803804); the Guangdong Province Science and Technology Planning Project (2017A030310124, 2017A050506041, 2017B030314166, 2019A1515010636, 2020A1515010607, and 2020B1111100006), the Guangdong Provincial Department of Education Project (2018KQNCX046), Guangzhou Science and Technology Project (202102020545) and the Guangdong Provincial Hospital of Chinese Medicine Special Fund (YN2018ZD08, YN2018HK01, YN2018RBA02, YN2016XP02, YN2019QJ04, and YN2019QJ08).

## Conflict of Interest

The authors declare that the research was conducted in the absence of any commercial or financial relationships that could be construed as a potential conflict of interest.

## Abbreviations

HUVECs, human umbilical vein endothelial cells; IMQ, imiquimod; MTX, methotrexate; VEGF, vascular endothelial growth factor; VEGFR, vascular endothelial growth factor receptor; ANG1, angiopoietin-1; HIF-1α, hypoxia-inducible factor-1 alpha; MAPK, mitogen-activated protein kinase; ERK, extracellular signal-regulated kinase; JNK, c-Jun N-terminal kinase; SOD, superoxide dismutase; GSH, glutathione; CAT, catalase; ROS, reactive oxygen species; MDA, malonaldehyde; LDH, lactate dehydrogenase.
